# Oxytocin as an Indicator of Psychological and Social Well-Being in Domesticated Animals: A Critical Review

**DOI:** 10.3389/fpsyg.2017.01521

**Published:** 2017-09-13

**Authors:** Jean-Loup Rault, Marleen van den Munkhof, Femke T. A. Buisman-Pijlman

**Affiliations:** ^1^Faculty of Veterinary and Agricultural Sciences, Animal Welfare Science Centre, University of Melbourne Parkville, VIC, Australia; ^2^Institute of Animal Husbandry and Animal Welfare, University of Veterinary Medicine Vienna, Austria; ^3^Faculty of Medicine, Utrecht University Utrecht, Netherlands; ^4^Adelaide Medical School, University of Adelaide Adelaide, SA, Australia; ^5^Robinson Research Institute, University of Adelaide Adelaide, SA, Australia

**Keywords:** affiliation, animal welfare, emotion, human-animal interaction, intranasal administration, oxytocin, positive, social behavior

## Abstract

Oxytocin is often portrayed as a hormone specific to social behavior, reflective of positive welfare states, and linked to mental states. Research on oxytocin in domesticated animal species has been few to date but is rapidly increasing (in dog, pig, cattle, sheep), with direct implications for animal welfare. This review evaluates the evidence for the specificity of oxytocin as an indicator of: 1. Social, 2. Positive, and 3. Psychological well-being. Oxytocin has most often been studied in socially relevant paradigms, with a lack of non-social control paradigms. Oxytocin research appears biased toward investigating positive valence, with a lack of control in valence or arousal. Oxytocin actions are modulated by the environmental and social contexts, which are important factors to consider. Limited evidence supports that oxytocin's actions are linked to psychological states; nevertheless whether this is a direct effect of oxytocin *per se* remains to be demonstrated. Overall, it is premature to judge oxytocin's potential as an animal welfare indicator given the few and discrepant findings and a lack of standardization in methodology. We cover potential causes for discrepancies and suggest solutions through appropriate methodological design, oxytocin sampling or delivery, analysis and reporting. Of particular interest, the oxytocinergic system as a whole remains poorly understood. Appreciation for the differences that social contact and group living pose in domesticated species and the way they interact with humans should be key considerations in using oxytocin as a psychosocial indicator of well-being.

## Introduction

Being able to display social behavior is key to the welfare of domesticated animals, who are all social species. Oxytocin (OT) has received increased attention for its involvement in the proximate mechanisms of social behavior, offering opportunities to elucidate the perception or processing of social stimuli.

This review aims to critically evaluate the validity and robustness of OT as an indicator of animal welfare related to the social environment. We focus on the primary literature on OT in domesticated animal species, because although OT has been well researched in human, non-human primates and rodents (Winslow et al., 2003; Neumann, 2009; Cavanaugh et al., 2016; Freeman and Young, 2016), an emerging theory is that domestication may have influenced the oxytocinergic system (Nagasawa et al., 2015), similarly to the effects of domestication on quantitative behavioral changes (Price, 2002). We discuss findings on the impact of experimental manipulations on endogenous OT concentration, differences in OT receptor gene polymorphisms and OT administration on behavior and human-animal interaction. We excluded the special case of maternal behavior, covered by previous reviews (Neumann, 2009; Kim and Strathearn, 2016), and studies of human-animal interaction focused on the human experience. This review does not intend to exhaustively cover the literature on OT in domesticated species but rather focuses on aspects relevant to behavior and welfare, highlighting findings and gaps in research. Approaches to study animal welfare and animal welfare assessment are covered elsewhere (Fraser, 2008; EFSA, 2012).

## Reflections on research to date

We found 32 relevant studies on OT in domesticated species, with 78% of them published in the last 5 years (Table 1). Studies used different methods: measures of central OT (in cerebrospinal fluid 3%, in brain sections 3%) or peripheral OT (in blood plasma or serum 31%, in urine 13%), administration of exogenous OT to study the animal's response (47%), study on brain OT gene expression (3%), and more recently OT receptor gene polymorphisms (13%), with 9% of studies using more than 1 approach. For studies that measured endogenous OT, 47% took a pre- and post-stimulus sample, 40% took repeated samples during the presentation of the stimulus, and 13% only took a sample at 1 time point (comparing between individuals). Only 22% of studies had a control group, whereas the rest used within-subject designs.

**Table 1:** Summary of studies on OT and social behavior in domesticated species to date.

			**Design**				**Measures**	
**Ref#**	**N & species (gender)**	**OT dose**	**Test design**	**Test category**	**Familiarity + with whom**	**Control condition**	**OT matrix measurement**	**Analysis method**
**ENDOGENOUS OXYTOCIN**								
Nagasawa et al., 2015	30 dogs; 11 wolves (m/f)	NA	OT pre and post: gazing, verbal, touch	Social (interspecies)	Familiar and unfamiliar person; voluntary contacts	Within	Urine (extracted)	RIA
Mitsui et al., 2011	9 dogs (m/f)	NA	food, water, exercise and touch	Social (interspecies)	Familiar human; imposed contacts	Within	Urine (extracted)	RIA
Rehn et al., 2014	12 dogs (f)	NA	OT during: physical or verbal contact, ignoring	Social (interspecies)/Isolation	Familiar human; imposed contacts	Within	Blood (non-extracted)	EIA
Pekkin et al., 2016	28 dogs (m/f)	NA	OT pre and post: pressure vest on effect noise stress	Social (interspecies)/Stress	Familiar human; voluntary contacts	Within	Urine (extracted)	ELISA
Odendaal and Meintjes, 2003	18 dogs (m/f)	NA	OT pre and post: touch, verbal, low-key play	Social (interspecies)	Familiar and unfamiliar person; imposed contacts	Within	Blood (non-extracted)	HPLC
Handlin et al., 2011	10 dogs (m)	NA	OT pre and post: touch verbal, ignore with female owners	Social (interspecies)	Familiar human; imposed contacts	Within	Blood (non-extracted)	EIA
Rault, 2016	5 pigs (f)	NA	OT pre and post: touch, verbal, positive and negative interaction with person	Social (interspecies)	Familiar human; voluntary contacts	Within	CSF (non-extracted)	ELISA
Bruckmaier et al., 1993	8 cows (f)	NA	OT pre and post: milking in different environments	Stress	Familiar vs. unfamiliar environment	Within	Blood (extracted)	RIA
Yayou et al., 2010, 2014, 2015	20 calves (f)	NA	OT repeatedly: sniffing, touching, mixing with unfamiliar conspecifics; during development	Stress/Novel environment/Social (intra-species)	Familiar and unfamiliar environment; familiar and unfamiliar conspecifics	Within	Blood (extracted)	EIA
Parrott and Thornton, 1989	10 sheep (m/f)	NA	OT pre and post: during isolation and in social environment; effect of opioid agonist and antagonist	Social (intra-species)/Stress	Familiar conspecifics and unfamiliar environment	Within	Blood	RIA
Coulon et al., 2013	16 lambs (f)	NA	OT during: touch, isolation, reunion	Social (interspecies)	Familiar human, voluntary or imposed contacts	Within	Blood	EIA
**OXYTOCIN ADMINISTRATION**								
Romero et al., 2014	16 dogs (m/f)	40 IU IN	OT pre and post: affiliation, proximity	Social (interspecies)/Social (intra-species)	Familiar human or dog	Within	Urine (extracted)	RIA
Oliva et al., 2016a	75 dogs (m/f)	24 IU IN	Ability to use experimenters' visual cues to find food; questionnaires	Social (interspecies)	Rating by familiar human, task with unfamiliar human	Within	NA	NA
Kovács et al., 2016	39 dogs (m/f)	12 IU IN	Spontaneous preference for biological motion versus non-biological control stimuli	Social (movement)	NA	Within	NA	NA
Hernádi et al., 2015	36 dogs (m/f)	12 IU IN	Response to threatening behavior owner or experimenter	Social (interspecies)/Stress	Familiar and unfamiliar human	Within	NA	NA
Oliva et al., 2015	62 dogs (m/f)	24 IU IN	Use of pointing and gazing cues by experimenter in object choice task	Social (interspecies)	Unfamiliar human	Within	NA	NA
MacChitella et al., 2017	17 dogs (m/f)	2 IU/kg IN	Use of pointing and gazing cues by experimenter in object choice task	Social (interspecies)	Unfamiliar human	Within	NA	NA
Kis et al., 2015	64 dogs (m/f)	12 IU IN	Pointing to find food in cognitive bias test	Social (interspecies)	Unfamiliar human	Between and Within	NA	NA
Romero et al., 2015	16 dogs (m/f)	40 IU IN	Social play between adult dogs	Social (intra-species)	Familiar conspecifics	Within	NA	NA
Rault et al., 2013a	24 piglets (m/f)	24 IU IN	Observe distress-related behavior during mixing with unfamiliar conspecifics	Social (intra-species)	Unfamiliar conspecifics	Between and Within	NA	NA
Camerlink et al., 2016	96 pigs (f)	24 IU IN	Observe social contact on return to pen after positive/negative/neural experience	Social (intra-species)	Familiar conspecifics	Between and Within	NA	NA
Rault et al., 2015	144 piglets (m/f)	24 IU IN/80 IU SC	Observe social behavior and food/water intake post-weaning;	Social (intra-species)/Stress	Familiar and unfamiliar conspecifics	Between	NA	NA
Reimert et al., 2015	96 pigs (f)	24 IU IN	Emotional contagion for positive and negative events: observing interaction	Social (intra-species)	Familiar conspecifics	Between and Within	NA	NA
Rault et al., 2013b	24 piglets (f)	24 IU IN	Behavior during isolation after prenatal stress or control	Stress	NA	Between	NA	NA
Rault, 2016	3 pigs (f)	36-60 IU IN	Endogenous OT collection overtime	Home pen normal environment	NA	Within	CSF (non-extracted)	ELISA
Mitsui et al., 2011	6 dogs (m)	24 × 10^−5^ IU IV in 4 bolus each 5 min	OT pre and post IV OT injection	Unfamiliar cages, non-social	NA	Within	Blood (non-extracted); urine (extracted)	RIA
Nagasawa et al., 2015	27 dogs (m/f)	40 IU IN	OT pre and post, dog behavior: gazing, touch, proximity	Social (interspecies)	Familiar and unfamiliar person	Within	Urine (extracted)	RIA
**OT RECEPTORS AND NEURONS**								
Oliva et al., 2016b	169 dogs and 12 wolves (m/f)	24 IU IN	Pointing and indicating by experimenter	Social (interspecies)	Unfamiliar human	NA	OTR	PCR
Kis et al., 2014	207 dogs (m/f)	NA	Greeting, threatening, separation with stranger and familiar person	Social (interspecies)	Familiar and unfamiliar humans	NA	OTR	PCR
Ottenheimer-Carrier et al., 2017	97 dogs (un)	NA	Personality questionnaire	Personality	NA	NA	OTR	PCR
Arahori et al., 2016	94 cats (m/f)	NA	Personality questionnaire	Personality	NA	NA	OTR	PCR
Guesdon et al., 2016	24 sheep (f)	NA	Isolation, presence, touch human	Social (interspecies)/Stress	Familiar human	Between	Neuronal activation PVN	Immunohistochemistry
Vellucci and Parrott, 1997	10 young pigs (m)	NA	Restraint	Stress	NA	Between	OT gene forebrain	Autoradiography

*m, male; f, female; un, unknown; NA, not applicable; IN, intranasal administration; SC, subcutaneous administration; Between, between-subject control; Within, within-subject control; OT, oxytocin; OTR, oxytocin receptor; Extracted, samples extracted prior to assaying; Non extracted, No mention made of extraction of sample prior to assaying; RIA, radioimmunoassay; EIA, enzyme immunoassay; HPLC, high-performance liquid chromatography*.

Average sample size was 48 subjects, ranging from 5 to 207 subjects. Most studies used dogs (53%: 44% mixed breeds and 9% single breed, and 6% with wolf as a comparison), then pig (22%), cattle (13%), sheep (9%), and cat (3%). Studies on dogs involved a variety of adult age dogs (over 12 months) whereas studies on farm animals involved mostly young, pre-pubertal weaned subjects. As mentioned earlier, we excluded studies of maternal behavior. Studies involved mixed sexes (56%, intact or castrated), females only (34%), males only (6%), or unreported (3%). The more pronounced effects of OT administration in females than males is well-recognized (Rault et al., 2013a; Nagasawa et al., 2015; Kovács et al., 2016; Oliva et al., 2016a), but whether sexes differ in endogenous OT remains unclear as the few studies that included both sexes did not report testing for a sex effect or individual OT profiles or had insufficient sample size.

The majority of OT studies were on human-animal interaction (51%), demonstrating that OT's function cross species boundaries. The rest was composed of intra-species interaction (22%) and social isolation (27%), with 24% of studies using more than 1 paradigm. Furthermore, a variety of experimental testing conditions have been used (Table 1).

The following sections explore the premise of OT as an indicator of social, positive and psychological well-being in domesticated animals based on the research to date and the main factors identified for the scope of this review. We discuss research from different animal species when available, but do not assume results should be similar across species given different ethological or evolutionary importance, which we refer to as species-specific social behavior.

## Are oxytocin's functions specifically social?

A large number of studies investigated the impact of social stimuli on endogenous OT concentration. A range of social settings trigger an OT response; the presence and magnitude of the response depending on a range of experimental factors such as familiarity of setting and partner, voluntary contact, and form of contact.

### Can only social variables influence OT release?

Studies generally showed OT increases in response to social interactions, but unfortunately few studies contained a non-social control situation. This is important because OT increases following stroking but also after exercising and eating in dogs (Mitsui et al., 2011) and OT increase in response to social and non-social stressors in rodents and humans (Nishioka et al., 1998; Olff et al., 2013). Conversely, OT did not differ between sheep kept in their groups *vs*. isolated in an unfamiliar environment (Parrott and Thornton, 1989). Furthermore, basal plasma OT relates to broader behaviors such as negative correlation with curiosity and general activity and positive correlation with fearfulness in dairy cows (Yayou et al., 2010, 2014).

### Impact of different types of social interaction

Visual contact with humans is sufficient to increase OT compared to isolation [dog: (Rehn et al., 2014), artifically-reared sheep: (Guesdon et al., 2016)], and there is a positive feedback loop between OT and gazing (i.e., visual contact) in dogs interacting with humans (Nagasawa et al., 2015). Nevertheless, additional physical contact increases OT for a longer duration (Rehn et al., 2014), and more frequent interactions initiated toward humans correlate with higher OT increase in CSF (Rault, 2016).

We propose that OT is released upon voluntary interaction by the animal rather than contact imposed on the animal, hence depending on the animal's control of the interaction. Indeed, OT was higher when interactions were reciprocated (Romero et al., 2014), whereas time spent near an owner asked to ignore the dog yielded inconsistent results, either correlating with (Pekkin et al., 2016) or with no effect on urine OT (Romero et al., 2014; Nagasawa et al., 2015). Furthermore, stroking imposed on the animal did not activate more OT neurons than human presence in hand-reared lambs (Guesdon et al., 2016), and did not increase plasma OT (Coulon et al., 2013).

In summary, social presence can trigger OT release, and physical contact intensify it, but further research is warranted to investigate whether OT release relates to species-specific social behavior and reciprocal interactions rather than contacts imposed on the subject.

### Impact of partner familiarity

Most human-animal interaction studies used familiar humans. The few studies that included familiar and unfamiliar humans suggest that OT's release is stimulated by familiar partners (Rehn et al., 2014; Hernádi et al., 2015; Nagasawa et al., 2015). Unexpectedly, OT administration reduced dog's friendliness toward their owner whereas it did not affect their response toward a stranger (Hernádi et al., 2015), but exogenous OT administration at supraphysiological levels causes OT to bind to vasopressin receptors, possibly resulting in confounded effects (Manning et al., 2012). Furthermore, in this last study, a stranger was standing behind them in the first situation versus their owner in the second situation. This may have influenced the dog's response because the stranger in their back may have provided a potential threat whereas their owner in the back social support, as dogs looked back more at their owner than the stranger (Hernádi et al., 2015). Overall, findings support that OT is involved with familiar rather than unfamiliar individuals (Bielsky and Young, 2004).

Conversely, in studies that used unfamiliar conspecifics, OT administration often increases negative social behavior and reduces positive social behavior (see Section Is Oxytocin an Indicator of Positive Valence? below). Social cognition is important in situations where animals need to determine whether the social partner is familiar or unfamiliar; an ability linked to oxytocin and vasopressin (Bielsky and Young, 2004).

### Summary on oxytocin and sociality

Oxytocin has most often been studied in socially relevant paradigms, but with a lack of non-social control paradigms to establish the specificity of OT to social contexts. It is difficult to disentangle it from a general stress coping mechanism in social species (Cavanaugh et al., 2016), in which OT may have evolved as the social arm of homeostatic processes (Buisman-Pijlman et al., 2014). Comparative studies using various species could help assess the relationship between OT and sociality. The presence of a partner increases OT release compared to social isolation, with a possible additional advantage of reciprocated contact, which requires further research with consideration of species-specific social behavior.

## Is oxytocin an indicator of positive valence?

In the quest for indicators of positive welfare states, OT is often proposed to reflect situations of positive valence. However, few studies have compared positive to negative or neutral situations. For instance, that urinary OT increases in three positive situations does not prove OT as a “biomarker of positive emotions” (Mitsui et al., 2011) unless a non-positive situation would have been included, although cortisol was included as a measurement of arousal.

Environmental context can modulate OT's actions. For instance, OT administration promoted positive social behaviors of dogs toward both their owners and familiar dogs (Romero et al., 2014), but reduced friendliness toward the owner in the presence of an approaching stranger, as discussed earlier (Hernádi et al., 2015). Opposite findings were found in pigs, in which OT administration in familiar groups reduced social contact in neutral or positive situations but increased it in negative situations (Camerlink et al., 2016). Conversely, CSF (endogenous) OT increased in pigs following positive human interaction, but not negative human interaction (Rault, 2016), although valence and familiarity of the partner were confounded.

The social context (e.g., partner familiarity) may also modulate OT's actions. Calves with high basal plasma OT postnatally showed higher social engagement, both affiliative and agonistic behaviors, in later life (Yayou et al., 2015), and exogenous studies showed that OT administration can increase aggression in pigs (Rault et al., 2013a, 2015). However, these studies involved animals mixed with unfamiliar conspecifics and in unfamiliar environments, i.e., stressful situations. Altogether, these findings are consistent with the in-group vs. out of group OT theory in humans (De Dreu, 2012), with OT's positive actions toward existing social partners and negative actions toward unfamiliar partners.

In summary, OT does not necessarily correlate with positive situations or outcomes. The OT literature appears biased toward investigating positive valence, with a lack of controlled paradigms for valence and arousal. There is evidence that negative situations also mobilize OT. We propose that OT may be evolutionarily linked to social coping strategies (Buisman-Pijlman et al., 2014; Cavanaugh et al., 2016), as the social arm of homeostatic processes, and as such neither positive nor negative but simply adaptive. The valence of OT's actions are modulated by the environmental and social contexts, and OT's theoretical function of preserving existing social bonds (Tops et al., 2014). Environmental and social factors are therefore important to consider in study design and interpretation (Olff et al., 2013).

## Are oxytocin's actions linked to specific psychological processes?

Oxytocin is often referred to as the “feel-good” hormone, or as an indicator of positive emotions (Mitsui et al., 2011). Rodent and human data highlight the effect of exogenous OT in increasing trust and reading of social cues, reducing anxiety and other psychological processes (Lee et al., 2009). There is no direct neurobiological evidence yet in domesticated species to support the role of OT in psychological, and particularly emotional, processes. Studies extrapolate their findings to psychological implications based on analogy with human studies (Mitsui et al., 2011). However, OT's role in human psychological processes is still debated (Nave et al., 2015). Particularly, whether the affective “feel good” effect is a direct or indirect effect of OT is unclear, given that OT antagonists do not block these effects (Uvnas-Moberg, 1998) and that the oxytocinergic system interact with other reward systems, notably opioidergic and dopaminergic systems that also increase in response to social interactions (Odendaal and Meintjes, 2003; Buisman-Pijlman et al., 2014; Tops et al., 2014) and impact on the HPA axis (Buisman-Pijlman et al., 2014; Tops et al., 2014).

Most of the knowledge in psychology is about the effect of intranasal OT administration, rather than correlative studies between endogenous OT and psychological states. Interestingly, OT administration induces a positive cognitive bias in dogs to ambivalent food cues (Kis et al., 2015).

The stage at which OT affects socio-cognitive processes currently debated in humans (perception vs. processing of social cues) has been followed up in dogs, with OT administration posited to reduce the attentional bias to social cues (Kovács et al., 2016), whereas others argue that OT does not alter perceptual salience of social cues or social anxiety but rather motivates social engagement (Romero et al., 2014).

The social motivation vs. social reward hypothetical functions, which appears in the human literature, is also relevant to domesticated animals. The hypothesis that OT increases social motivation is supported by exogenous OT studies, with dogs administered OT initiating more contact toward a familiar dog and owner (Romero et al., 2015), even when owners were instructed to ignore or only briefly reciprocate (Romero et al., 2014; Nagasawa et al., 2015). The hypothesis that OT conditions the rewarding value of social cues is supported by endogenous OT studies, where the failure from humans to reciprocate contact results in lower plasma OT concentration over time compared to the initial reunion (Rehn et al., 2014), but no change in urine OT (Nagasawa et al., 2015). More frequent measurements of OT over time could allow discerning appetitive from consummatory motivations.

In summary, there is currently limited evidence that OT's actions are linked to psychological states. Nevertheless, it remains to be demonstrated that it is a direct effect of OT *per se*. This is a worthwhile area of research given the increasing interest in affective states (feelings, emotion, and cognition) in psychology and animal welfare science.

## Potential and current limitations of oxytocin as an animal welfare indicator

While findings are coming at a quick pace, the few and discrepant findings make it premature to conclusively decide on OT's potential as an animal welfare indicator.

### Oxytocin's potential as an animal welfare indicator

An animal-based indicator of welfare should be valid and robust (EFSA, 2012). The interpretation of OT as an animal welfare measure requires precise and consistent results. Unfortunately, we highlighted above substantial inconsistencies in findings to use OT as a welfare indicator, possibly due to the exploratory stage of the research. Possible causes of discrepancy are highlighted in Table 2, along with potential solutions. Full reporting of the factors listed in Table 2 would enhance rigor in OT research while abiding by good scientific practices. Standardization of the experimental testing procedures may also help to compare findings, as is commonly done for research on primates and rodents.

**Table 2:** Summary of common research design and methodological pitfalls, and potential solutions to enhance validity and comparison in OT research.

**Factor**	**Problems**	**Potential solutions**
Sample size	Low number of subjects	Use power analysis to calculate sample size^a^
	Heterogenous sample: e.g., breed, age, previous experience, sex, hormonal status	Minimize the number of variables between subjects and situations
	High inter-individual variability	Adopt a within-subject design
Testing paradigm	Sole testing paradigm	Use more than 1 paradigm, adapted to the hypothesis (e.g., social vs. non-social; positive vs. negative valence) to determine the specificity of the findings
	No control treatment	Include control group (between-subject design)
	Unknown contextual effects	Adopt a counterbalanced design
	Lack of standardization or measure of (social) stimulus	Standardize the stimulus, or measure covariates to take into account at the data analysis stage
	Too few methodological details	List individual (current characteristics and past experiences) and context description in the methodology to improve content validity of findings. Choose behavioral test and conditions that are species-appropriate; choose settings to fit aim: either familiar or unfamiliar environment/person/animals and control for it
OT sample collection^b^	Different sampling matrices (e.g., plasma, urine, CSF)	Study the correlation between OT in different matrices and biological actions/targets
	Inappropriate time-point for sample collection	Timepoint appropriate to OT release and half-life in the matrix; prefer multiple time-points if possible to assess OT dynamics overtime
	Varying collection procedures (OT is a peptide hormone sensitive to degradation, especially by freeze-thaw cycles)	Uniformization of collection procedures within study, researchers blind to experimental treatments
OT sample analysis: bioanalytic validity and reliability*^2^*	Sensitivity	Demonstrate that concentration falls within the assay detection limit
	Precision and reliability	Determine intra- and inter-assay CVs in your lab
	Accuracy	Demonstrate quality control steps: e.g., spiking, linear dilution; correlation between analysis technique used and other validated techniques, or cite peer-reviewed published validation
	Specificity	Compare extracted vs. unextracted samples; report cross-reactivity or cite published validation
OT administration	Route of administration	Consider the mode of delivery: subject position, subject habituation and administrator training, product additives, concentration/volume, absorption and clearance rate^c^
	Dose	Assess dose-dependent response through a pilot trial or within the main experiment; aim for minimal dose; administer OT and a selective antagonist
	Timeline for testing post-administration	Use multiple sampling timepoints if possible; time of day
Study replication	Lack of study replication	Use multiple replicates within a study; replicate studies from other researchers
Results analysis	Failure to report initial OT concentration data (“absolute” OT concentrations) or reporting solely correlation	Report absolute concentrations, supplementary file to share large dataset, especially interesting for individual data profile and variation
	Use of incorrect statistical analysis	Correct for multiple comparisons, baseline data, etc
	Omitting or discarding data	Identify causes for outliers, justify the treatment of outliers
Publication of findings	Large bias toward positive over null findings^d^	Lay out the soundness of the experimental design and proper analysis of the findings^e^

a See http://www.3rs-reduction.co.uk/html/6__power_and_sample_size.html

b *For instance (Robinson et al., 2014)*.

c *For instance (Guastella et al., 2013)*.

d *For instance (Lane et al., 2016)*.

e *For instance (Kilkenny et al., 2010)*.

Briefly, OT is a peptide hormone, which makes it especially sensitive to sampling collection procedures and analytic methods compared to steroid hormones like cortisol. Given OT's variability between individuals and contexts (Olff et al., 2013), within-subject experimental designs (see Kekecs et al., 2016) and counterbalanced designs should be favored to tackle contextual modulation. Inter-individual variation is a well-known phenomenon in OT research, and worthy data to report (individual data profile can be shared through Supplementary Material, see for instance (Nagasawa et al., 2015)), to help further studies and meta-studies progress our understanding of the OT system's response and actions. The reproducibility crisis of science does not spare OT research (Nave et al., 2015), and we found only one study replication (MacChitella et al., 2017).

### Overlooked areas of oxytocin research

The biological significance of OT measured in different matrices (e.g., centrally but also blood, urine, saliva, and milk) remains to be elucidated. The function of the oxytocinergic system as a whole is poorly understood, and most studies focused solely on its circulating hormone (through measurement or administration), rather than OT-secreting neurons or the OT receptor (Freeman and Young, 2016). Oxytocin receptor gene polymorphisms have provided insights into variation in human-animal interaction. Nevertheless, the role of genetic (breed) and epigenetic (rearing) factors remain to be clarified, as the OT receptor gene differs between wolf and dogs (Oliva et al., 2016b) but differences between animals that vary in their sociality returned positive [dogs: (Kis et al., 2014); cats: (Arahori et al., 2016)] or null findings [dogs: (Oliva et al., 2016b; Ottenheimer-Carrier et al., 2017)].

The drawbacks of sampling endogenous OT explain the attractiveness of intranasal OT administration, boosted by pioneering studies in humans (Born et al., 2002; Kosfeld et al., 2005). However, OT dose-response studies are lacking, species-specific metabolic differences in absorption or clearance rate are unknown, and the use of selective OT antagonists would strengthen the evidence for OT-mediated pathways (Guastella et al., 2013; Cavanaugh et al., 2016). For instance, most studies test animals 45 min post-OT administration following human studies, but effects may vary between sampling matrices or species (Mitsui et al., 2011; Nagasawa et al., 2015; Rault, 2016). The biological relevance of commonly administered OT doses is also questionable, as intranasal administration of 36–60 IU increased endogenous CSF OT 20- to 60-fold in pigs (Rault, 2016), well-beyond normal physiological concentrations, although plasma OT increases appear to be only three-fold higher than baseline in dogs after delivery of 40 IU, and to a lower extent but inconsistently in urine (Romero et al., 2014). This also raises the likelihood of activating the vasopressinergic system by OT administration, resulting in potential confounding behavioral effects (Manning et al., 2012). Interestingly, dogs with lower endogenous OT concentrations were more responsive to exogenous OT administration than dogs with higher endogenous OT concentrations (Romero et al., 2014).

The responsiveness of the OT system (synthesis, pulsatile release, receptor numbers, and binding) to stimuli remains poorly understood, especially as most studies only sampled at a couple of timepoints. Studying OT's role along with complementary physiological systems (vasopressinergic, opioidergic, dopaminergic, and the HPA axis) is also crucial to comprehend OT's function.

The potential modulation of the OT system through development and experience (Buisman-Pijlman et al., 2014), and particularly its epigenetic bases, warrant further research. For instance, basal plasma OT related to behavioral traits in the neonatal calves (Yayou et al., 2010) but not with their behavior in later life (Yayou et al., 2014) or only in specific conditions (Yayou et al., 2015). There is a crucial lack of knowledge of the ontogeny of the oxytocinergic system in domesticated species.

### Oxytocin and social communication

There is an increasing body of evidence that OT mediates social communication and social cognition, particularly using human-dog interaction as a model (Nagasawa et al., 2015; Kovács et al., 2016). Oxytocin administration enhances dogs' performance using human momentary distal pointing cues (Oliva et al., 2015; MacChitella et al., 2017), increases gaze to owner (Nagasawa et al., 2015), decreases aversion to unfamiliar human gaze (Oliva et al., 2015), but also block the ability of owner to predict the performance of their dog (Oliva et al., 2016a). The stage at which OT intervenes in socio-cognitive processes remains unclear (see Section Are Oxytocin's Actions Linked to Specific Psychological Processes?).

Intriguing evidence suggests that OT administration may not only influence the treated animal, but also non-treated conspecifics in the same environment. For instance, OT administration to a pig altered the behavior of a conspecific unable to see the OT-administered pig, reducing defecation during the negative situation and reducing low tail during the positive situation (Reimert et al., 2015). Similarly, OT administration affected cage mates through olfactorily-mediated stress inhibiting effects in rats (Agren and Lundeberg, 2002) and in humans (Weisman et al., 2012).

The involvement of OT in social communication promises to be a fascinating area of research, while emphasizing the need to monitor complementary measures such as behavior and vocalization.

## Implications: can oxytocin be trusted as an animal welfare indicator?

Focusing on the biological significance of OT in the regulation of psychological and behavioral states may help reconcile findings. A greater understanding of the effects of genetic, epigenetic and ontogeny on the oxytocinergic system is highly relevant to domesticated animals. Accumulating evidence in other species also shows that OT's actions are moderated by context and inter-individual differences. This is determinant to the use of OT as an animal welfare indicator sensitive to the state of interest and robust to extraneous factors. Furthermore, classic parameters for animal welfare measures such as sensitivity, specificity, and repeatability remain to be tested. Indeed, research on OT in domesticated species brings the advantage of potentially well-controlled experiments. It also has direct implications for animal welfare given the importance of social factors and the ability for human management practices to include situations conducive to OT system's development and stimulation.

## Author contributions

Mv screened the existing literature and drafted Table 1. JR and FB analyzed and interpreted the literature database and wrote the draft of the manuscript. JR, Mv, and FB reviewed and approved the final manuscript.

### Conflict of interest statement

The authors declare that the research was conducted in the absence of any commercial or financial relationships that could be construed as a potential conflict of interest. The handling Editor declared a shared affiliation, though no other collaboration, with one of the authors JR and states that the process nevertheless met the standards of a fair and objective review
